# Modeling the Impact of Lesions in the Human Brain

**DOI:** 10.1371/journal.pcbi.1000408

**Published:** 2009-06-12

**Authors:** Jeffrey Alstott, Michael Breakspear, Patric Hagmann, Leila Cammoun, Olaf Sporns

**Affiliations:** 1 Program in Cognitive Science, Indiana University, Bloomington, Indiana, United States of America; 2 Queensland Institute of Medical Research, Brisbane, Australia; 3 Royal Brisbane and Women's Hospital, Brisbane, Australia; 4 School of Psychiatry, University of South Wales, Sydney, Australia; 5 The Black Dog Institute, Sydney, Australia; 6 Signal Processing Laboratory 5, Ecole Polytechnique Fédérale de Lausanne, Lausanne, Switzerland; 7 Department of Radiology, University Hospital Center, University of Lausanne, Lausanne, Switzerland; 8 Department of Psychological and Brain Sciences, Indiana University, Bloomington, Indiana, United States of America; University College London, United Kingdom

## Abstract

Lesions of anatomical brain networks result in functional disturbances of brain systems and behavior which depend sensitively, often unpredictably, on the lesion site. The availability of whole-brain maps of structural connections within the human cerebrum and our increased understanding of the physiology and large-scale dynamics of cortical networks allow us to investigate the functional consequences of focal brain lesions in a computational model. We simulate the dynamic effects of lesions placed in different regions of the cerebral cortex by recording changes in the pattern of endogenous (“resting-state”) neural activity. We find that lesions produce specific patterns of altered functional connectivity among distant regions of cortex, often affecting both cortical hemispheres. The magnitude of these dynamic effects depends on the lesion location and is partly predicted by structural network properties of the lesion site. In the model, lesions along the cortical midline and in the vicinity of the temporo-parietal junction result in large and widely distributed changes in functional connectivity, while lesions of primary sensory or motor regions remain more localized. The model suggests that dynamic lesion effects can be predicted on the basis of specific network measures of structural brain networks and that these effects may be related to known behavioral and cognitive consequences of brain lesions.

## Author Summary

Every year, millions of people suffer the consequences of brain damage, as a result of stroke, traumatic brain injury, cancer or degenerative brain disease. The cognitive and behavioral symptoms of focal lesions of the brain are highly variable and in many cases depend on the location of the lesion site. Can we predict the functional impact of such lesions on the basis of a computational model of the brain's structure and dynamics? Numerous other systems that form complex networks have been analyzed for their vulnerability to structural damage. In many cases, the degree to which such systems are perturbed depends on network attributes of the deleted nodes and connections. We apply this network approach to investigate the structural and functional impact of localized lesions of a model of the cerebral cortex. When we delete nodes that occupy, in the intact brain, a highly central position, we find that the dynamic interactions between nodes in the remaining brain are greatly disturbed. In contrast, deletion of less central nodes has relatively little effect. In the model, some of the most disruptive lesion sites correspond to locations in the brain where lesions produce complex cognitive disturbances. Our modeling approach aims towards linking disturbances of structural brain networks to specific clinical outcomes.

## Introduction

Recent advances in noninvasive imaging technology have allowed the creation of comprehensive whole-brain maps of the structural connections of the human cerebrum [1]–[7]. These maps have led to the quantitative characterization of various aspects of the network architecture of the brain, including degree distributions, small-world attributes, centrality and modularity. Comparative studies of structural and functional connectivity indicate that the presence of structural links between pairs of cortical regions is predictive of the occurrence of endogenously driven (resting-state) functional connectivity [4],[8],[9]. The mapping of structural connectivity has also enabled the construction of computational models of resting state activity [10],[11]. The direct comparison of empirically observed and computationally modeled resting state functional connectivity revealed a high degree of overlap, supporting the idea that large-scale structural brain networks do indeed shape and constrain endogenous patterns of functional connectivity [8].

The structural or functional robustness of networks has been investigated in a number of complex systems [12],[13], including biological networks [14]–[16] In the case of the brain, acute injuries from trauma, tumor, or stroke, as well as chronic or degenerative disturbances due to disease, correspond to node and edge deletions in the structural brain network. Many of the cognitive and behavioral effects of brain lesions are highly variable and their mechanistic origins remain difficult to discern. Nevertheless, lesions of specific brain regions are often associated with specific cognitive and behavioral disturbances, and lesions of some areas tend to have more severe effects than others [17]–[19]. Vulnerability analyses [20]–[24] of several non-human primate cortical networks suggest that lesion effects show regional specificity as well as non-local and distributed effects.

We describe a model of lesion effects in the human brain, based on a previously published map of structural connections [4] and a biophysical model of endogenous neural dynamics [8]. We investigate the effects of focal lesions (removing a spatially localized set of nodes and connections) on the endogenous dynamics of the remaining brain. We identify structural measures of brain connectivity that are predictive of the magnitude of the perturbations in the endogenous neural dynamics. We discuss our results in light of known behavioral and cognitive lesion effects. The computational and complex network approach taken in this paper provides a new link between localized structural damage of brain networks and global disruptions of dynamic interactions.

## Methods

### Connectivity Data Set

The structural connectivity (SC) data set used in the present paper is identical to the one described and displayed in ref [8], based on diffusion MRI data first described in ref [4]. Briefly, structural connections were derived from diffusion spectrum imaging (DSI) of five healthy right handed male participants. The segmented cortical gray matter was partitioned into 66 anatomical regions according to anatomical landmarks using Freesurfer (surfer.nmr.mgh.harvard.edu) and 998 regions of interest (ROIs). The 998 ROIs were chosen to provide a roughly uniform tiling of the cerebral cortex (each ROI∼1.5 cm^2^) so that their borders aligned with those of the 66 anatomical regions. White matter tractography was performed with a custom streamline algorithm and fiber connectivity was aggregated across all voxels within each of the 998 predefined ROIs. The fiber strengths produced by the streamline tractography algorithm were exponentially distributed and spanned several orders of magnitude. Since connection weights in our model are meant to express physiological efficacy rather than fiber counts or the thickness of fiber tracts, we resampled the raw fiber strengths into a Gaussian distribution with a mean of 0.5 and a standard deviation of 0.1 dimensionless units. This transformation does not alter the rank-ordering of strong to weak pathways, but it compresses the scale of physiological efficacies (connection strengths). We created an “average SC matrix” from the resampled connection maps of individual participants. In this average SC map, structural connections were deemed absent overall, i.e. set to zero, if they were absent in more than 3 participants.

### Modeled Neural Dynamics

Neuronal dynamics were simulated using a system of neural masses coupled to one another with strengths linearly proportional to the resampled fiber strengths at each edge. Each neural mass represents a population of densely interconnected excitatory and inhibitory neurons, in which the effects of both ligand- and voltage-gated membrane channels are accounted for. This model was first developed in [25] and has previously been employed in an anatomically-informed model of large-scale functional connectivity in the macaque monkey [10] as well as for modeling human resting-state functional connectivity [8]. The model was simulated in Matlab R2007a (Mathworks, Natick, MA) at a time resolution of 0.2 msec. Before data analysis, resulting data sets are downsampled to a time resolution of 1 millisecond. After an initial transient of 2 minutes which was discarded, runs proceeded for a total of 8 minutes. Simulated BOLD signals were computed by using a nonlinear hemodynamic model as previously described [8],[10],[26]. While all simulations were carried out with the same set of haemodynamic parameters, future studies may incorporate individual variations, e.g. to take into account effects of disease state on blood vessel compliance, or regional variations of the haemodynamic response across different brain regions. Cross-correlation matrices of BOLD time-series (functional connectivity, FC) were derived without regressing out the global signal average, as this procedure may affect correlation pattern and magnitude. For each lesion, as well as for unlesioned controls, we conducted five simulation runs starting from random initial conditions. Data analyses were carried out on correlation matrices averaged over these five runs. For more details see refs. [8],[10],[25].

### Lesions

The structural connectivity matrix was lesioned in two ways: sequential single node deletions and localized area removal. The first method was aiming at a structural failure analysis, and included both “random” and “targeted” node deletions, involving the sequential removal of nodes (ROIs), one by one, until the network had shrunk to a single remaining node. For random node removal, we removed a single randomly chosen node at each step. This process was repeated 25 times. For targeted node removal, we first computed the node degree (defined as the number of connections at each node), node strength (defined as the sum of all the weights of the connections at each node) or the node betweenness centrality [27] for all nodes in the network. Then we removed the single node with the highest degree, strength or centrality. Degree, strength and centrality were then re-computed and the next node was selected for removal, until one last node remained. At each step during random and targeted node removal we calculated several structural network measures, including the size of the largest connected component of the remaining network and the global efficiency. Global efficiency is computed as the average of the inverse distance between all nodes and captures the network's capacity for communication along short paths [28].

**Figure 1:**
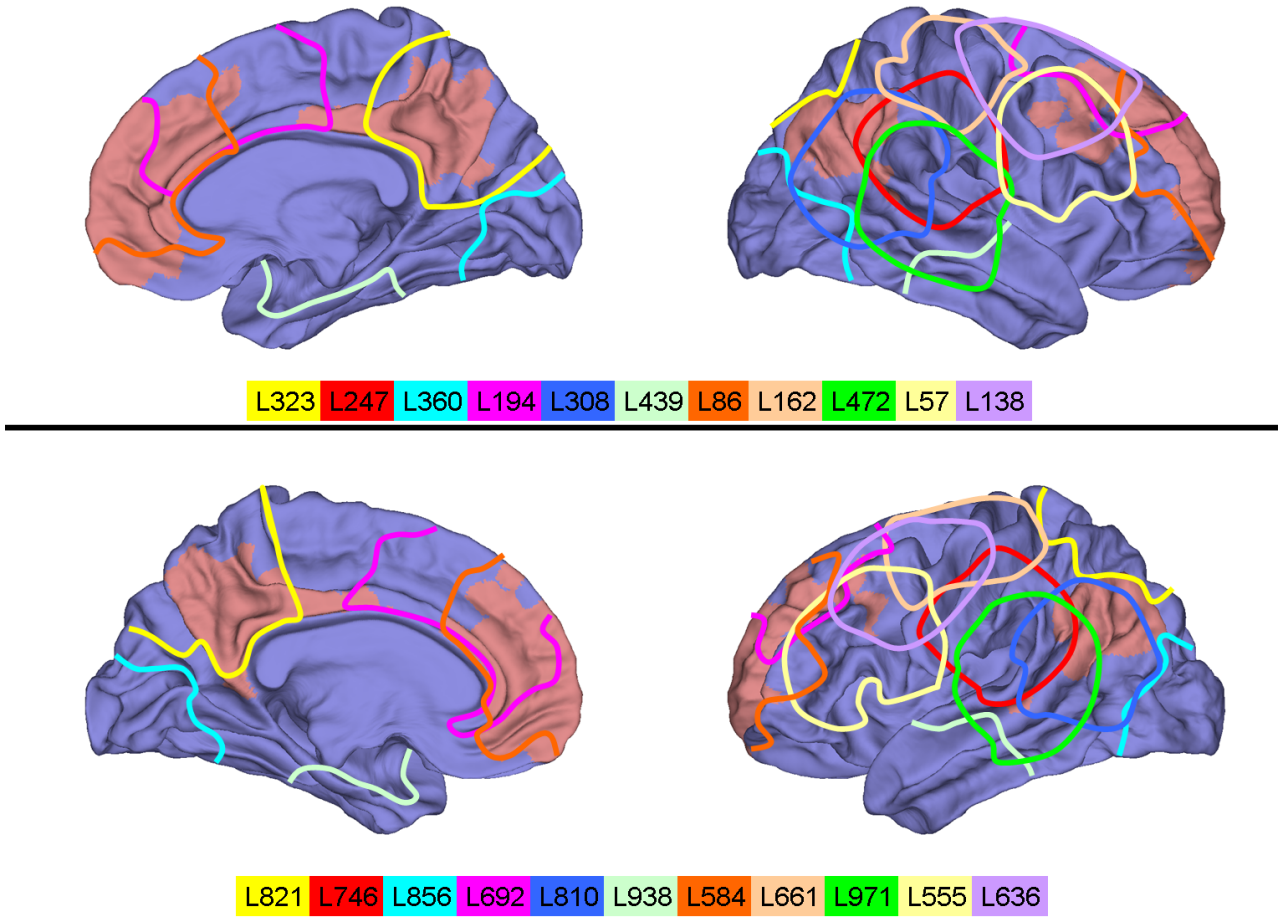
Lesion locations. Diagrams show a rendering of a standard cortical surface, with ROIs that form part of the DMN indicated in light red. Outlines indicate approximate lesion locations. All lesions are comprised of 50 ROIs. Lesion labels correspond to lesion names in Table 1 and 2.

The second lesion type, localized lesions, was aiming at dynamic and functional failure analysis. These lesions were carried out by removing all nodes and their connections within a spatially defined region around a central location. The central location was defined by a standard x,y,z Talairach coordinate and a fixed number of ROIs closest to this central location were deleted. Closeness was determined by the Euclidean distance. Lesions involved the deletion of nodes (“gray matter”) and their afferent/efferent connections only – we did not attempt to model “white matter” volume, for example by including lesions of “fibers of passage”. Computational considerations prevented us from simulating lesions centered on all 998 ROIs, and from varying the lesion extent. We selected a lesion size of 50 ROIs, corresponding to about 5% of the cortical surface, which was large enough to have significant effects on neural dynamics, and small enough to preserve the regional specificity of the lesions. A complete list of all lesions, their central locations, spatial coordinates, and affected anatomical subregions are provided in Table 1. The spatial location and extent of all lesions is depicted in Figure 1. Jointly, all lesions described in this paper cover about 80 percent of the cortical surface. Figure 1 also illustrates the relation of all lesions to the default mode network (DMN). The DMN was comprised of 200 ROIs which had earlier been determined from empirical fMRI studies [8], and contained portions of the precuneus/posterior cingulate cortex, medial and superior frontal cortex, and lateral parietal cortex.

**Table 1:** Modeled lesions and lesion locations.

	Right Hemisphere				
	Lesion name	ROI center	Talairach coordinate	Center region	Lesioned regions
Cortical midline	L323	323	(6, −56, 38)	rPCUN	rCUN, rISTC, rPCUN
	L194	194	(5 16 31)	rCAC	rCAC, rCMF, rSF
Parietal and temporal cortex	L308	308	(47 −51 22)	rIP	rBSTS, rIP, rSMAR
	L247	247	(62 −31 28)	rSMAR	rPSTC, rSMAR, rTT
	L472	472	(65 −32 10)	rST	rBSTS, rMT, rST, rSMAR, rTT
	L439	439	(50 −11 −29)	rIT	rENT, rIT, rST, rTP
Frontal cortex	L86	86	(7 48 21)	rSF	rCAC, rFP, rRAC, rRMF, rSF
	L138	138	(39 9 51)	rCMF	rCMF, rPREC
	L57	57	(40 9 21)	rPOPE	rCMF, rPOPE
Sensory, motor	L360	360	(26 −94 −6)	rLOCC	rLOCC, rLING, rPCAL
	L162	162	(34 −23 46)	rPREC	rPSTC

	Left Hemisphere				
	Lesion name	ROI center	Talairach coordinate	Center region	Lesioned regions
Cortical midline	L821	821	(−8 −57 47)	lPCUN	lISTC, lPCUN, lSP
	L692	692	(−7 26 26)	lCAC	lCAC, lRAC, lSF
Parietal and temporal cortex	L810	810	(−45 −50 20)	lIP	lBSTS, lIP
	L746	746	(−58 −25 28)	lSMAR	lPSTC, lSMAR
	L971	971	(−61 −36 12)	lST	lBSTS, lMT, lSMAR, lTT
	L938	938	(−44 −10 −26)	lIT	lENT, lIT, lMT, lPARH, lST, lTP
Frontal cortex	L584	584	(−8 52 17)	lSF	lCAC, lFP, lRAC, lRMF
	L636	636	(−39 7 42)	lCMF	lCMF, lPREC
	L555	555	(−42 22 18)	lPOPE	lCMF, lPOPE, lPTRI, lRMF
Sensory, motor	L856	856	(−25 −93 −7)	lLOCC	lLOCC, lLING, lPCAL
	L661	661	(−34 −9 52)	lPREC	lPREC

Lesions are named after the number of the central ROI and all lesions comprise a total of 50 ROIs. “Center region” refers to the name of the anatomical subdivision to which the central ROI belongs. “Lesioned regions” lists all anatomical subdivisions that are removed by at least 50% or their constituent ROIs. Anatomical subdivisions are named as follows: each label consists of two parts, a prefix for the cortical hemisphere (r = right hemisphere, l = left hemisphere) and one of 33 designators: BSTS = bank of the superior temporal sulcus, CAC = caudal anterior cingulate cortex, CMF = caudal middle frontal cortex, CUN = cuneus, ENT = entorhinal cortex, FP = frontal pole, FUS = fusiform gyrus, IP = inferior parietal cortex, IT = inferior temporal cortex, ISTC = isthmus of the cingulate cortex, LOCC = lateral occipital cortex, LOF = lateral orbitofrontal cortex, LING = lingual gyrus, MOF = medial orbitofrontal cortex, MT = middle temporal cortex, PARC = paracentral lobule, PARH = parahippocampal cortex, POPE = pars opercularis, PORB = pars orbitalis, PTRI = pars triangularis, PCAL = pericalcarine cortex, PSTS = postcentral gyrus, PC = posterior cingulate cortex, PREC = precentral gyrus, PCUN = precuneus, RAC = rostral anterior cingulate cortex, RMF = rostral middle frontal cortex, SF = superior frontal cortex, SP = superior parietal cortex, ST = superior temporal cortex, SMAR = supramarginal gyrus, TP = temporal pole, TT = transverse temporal cortex.

All graph-theoretical measures (path length, centrality, efficiency) reported in this study were computed from a structural network that preserved edge weights, as previously described [4].

### Measures of Lesion Effects

**Figure 2:**
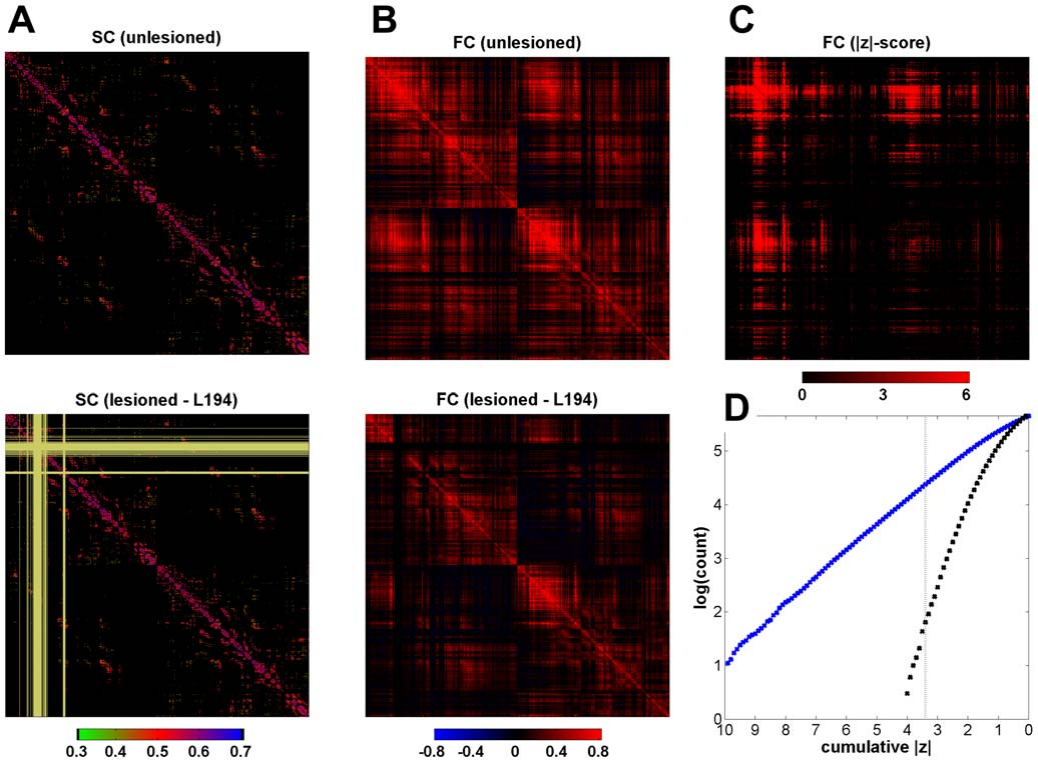
Structural connectivity, functional connectivity, and measurement of lesion effects. (A) Top: Intact “unlesioned” structural connectivity (SC). Bottom: lesioned SC. The lesion shown here is L194 and the lesioned portion of the matrix is indicated in light yellow. (B) Top: Unlesioned functional connectivity (FC) matrix, obtained after averaging BOLD cross-correlations from 5 simulation runs. Bottom: lesioned FC matrix (L194), averaged over 5 runs. (C) z-score matrix after subtraction of normalized cross-correlations. (D) Cumulative distribution of z-scores of functional connections after subtraction of lesioned (L194) from unlesioned FC (blue dots) and after subtraction of two sets of 5 unlesioned runs (black dots). The dashed line marks z = 3.3, and the number of functional connections at this threshold was taken as one measure of lesion impact.

The nature of the computational model does not allow us to probe directly for behavioral or cognitive lesion effects. Thus, our measures of lesion effects are confined to estimates of the lesion's immediate structural and dynamic impact. Examples of structural (SC) and BOLD cross-correlation matrices (FC) before and after a lesion are shown in Figure 2. Lesion effects were quantified in several ways, all of which produced similar patterns of results (Table 2). The distance between the unlesioned and lesioned FC matrix was calculated as
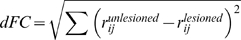
Where r_ij_ is the functional connectivity measure (cross-correlation) between nodes i and j. This distance dFC was computed for both the high-resolution FC matrices (998 ROIs) and for the regionally averaged FC matrix (66 regions). We computed two distances, one of which included functional connections of all ROIs (dFC), while the other only measured the distance between ROI pairs that were not involved in the lesion itself (dFC′).

**Table 2:** Magnitude and pattern of dynamic lesion effects.

	Right Hemisphere						
	Lesion name	Magnitude of Lesion Effects		Pattern of Lesion Effects			
		z′	top 50%	RH>LH	CC>(RH+LH)	DMN>non-DMN	W>S
Cortical midline	L323	8694	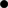		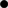		
	L194	26384	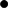	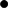	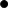	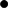	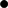
Parietal and temporal cortex	L308	2636		•	•		•
	L247	830		•			•
	L472	11253	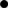	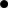			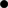
	L439	1369		•	•		•
Frontal cortex	L86	21448	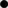		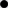	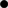	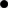
	L138	9255	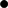	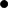	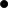	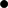	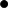
	L57	7077	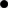	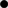		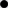	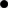
Sensory, motor	L360	1621		•	•		•
	L162	1851		•			•

Lesions are tabulated as in Table 1. Magnitude of lesion effects measures: z′ = sum of all significantly altered functional connections (|z|>3.3), excluding functional connections of lesioned nodes; top 50% = lesions whose z′ is in the top half. Pattern of lesion effects measures: RH>LH, LH>RH = number of significant functional connections in the left versus right cerebral hemisphere; CC>(RH+LH) = greater number of significant cross-hemispheric versus intra-hemispheric functional connections; DMN>non-DMN = greater proportion of significantly changed functional connections at ROIs that are part of the DMN versus ROIs that are not part of the DMN; W>S greater number of significantly weakened versus significantly strengthened functional connections; 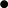 = yes (large-effect lesion); • = yes (small-effect lesion).

A second way to measure the difference between two correlation matrices was computed as follows. First, we converted the two correlation matrices (before and after lesioning) to a normal distribution by using Fisher's z-transform. To test the hypothesis that the two sets of correlations were drawn from different distributions we computed z-scores, according to
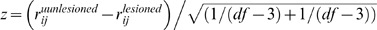
where df corresponds to the effective degrees of freedom. The value for df was estimated following procedures used for analyzing empirically obtained correlation matrices (e.g. ref [29]). Using a correction factor for independent frames (estimated according to Bartlett's theory [30]) of 3, and computing correlations from 5 independent runs of 8 minutes each, with 30 data samples/minute, we obtained df = 400. We then counted the number of functional connections that exceeded a significance threshold of |z|>3.3. To test the validity of this threshold we compared two correlation matrices computed from independent sets of 5 unlesioned runs against each other. After normalization, z-score transformation and thresholding at |z|>3.3, we detected 91 false positives out of nearly 500,000 comparisons (Figure 2D), indicating that the error rate is p<0.001. We concluded that for simulations of lesions the occurrence of a large number of functional connections with |z|>3.3 reflected specific lesion effects with very high probability. Choosing higher thresholds (e.g. |z|>5) did not affect the main conclusions of the study (data not shown).

## Results

**Figure 3:**
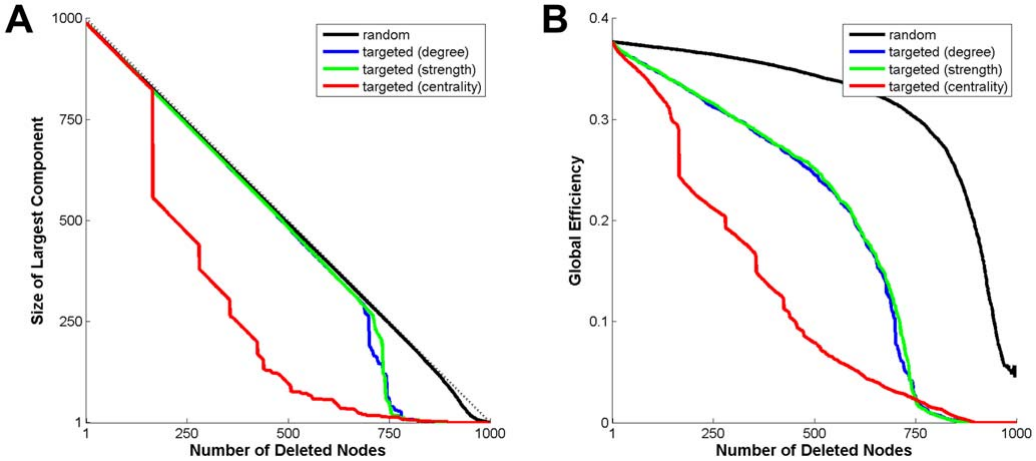
Analysis of robustness on the basis of random/targeted node deletions. The plots show the size of the largest network component (A) and the global efficiency (B) as a function of the number of deleted nodes. The curve for random node deletion is an average of 25 different random sequences. The other three curves represent unique sequences of node deletion determined by node degree (blue) strength (green) or node centrality (red).

Several previous studies have examined the direct effects of node deletions on network structure and connectivity. Thus, we first examined the effects of random and targeted node removal on the structural integrity of the network, measured as the size of the largest connected component (Figure 3). Random removal of nodes did not affect network integrity until almost all of the nodes had been deleted. Targeted removal of nodes on the basis of node degree or node strength disconnected the network only after approximately three quarters of all nodes had been deleted. In contrast, targeting nodes on the basis of their centrality resulted in the appearance of disconnected components after deletion of only 164 nodes. Targeting highly central nodes also resulted in a rapid decrease in the network's global efficiency, while targeted removal of nodes with high degree or high strength resulted in a more gradual decline in efficiency.. We performed identical analyses on a set of control networks whose global topology had been randomized while preserving the sequence of node degrees. These randomized controls were highly resilient to removal of nodes based on centrality or strength, remaining strongly connected until more than 700 nodes had been deleted (results not shown). These results indicate that the structural network is relatively insensitive to random node deletion, or to node deletion targeting nodes according to their degree or strength, while showing much greater vulnerability to targeted node deletion on the basis of centrality.

The potential dynamic effects of focal brain lesions on neural activity have remained relatively unexplored. Here, we compared functional connectivity patterns due to endogenous neural dynamics before and after a lesion was made. Despite equal lesion size (50 nodes) dynamic lesion effects exhibited marked differences depending on lesion location. These differences involved both the magnitude and the spatial pattern of changed functional connections (Table 2). Posterior and anterior lesions along the cortical midline, as well as a subset of lesions in frontal, parietal and temporal cortex, had extensive effects. With few exceptions lesion effects were stronger in the ipsilateral hemisphere, and mostly involved weakening of functional coupling. Lesions closer to the midline tended to be more disruptive of cross-hemispheric coupling than more lateral lesions. A subset of lesions in frontal cortex and in the anterior cingulate had disproportionately strong effects on functional connections involving the default mode network.

**Figure 4:**
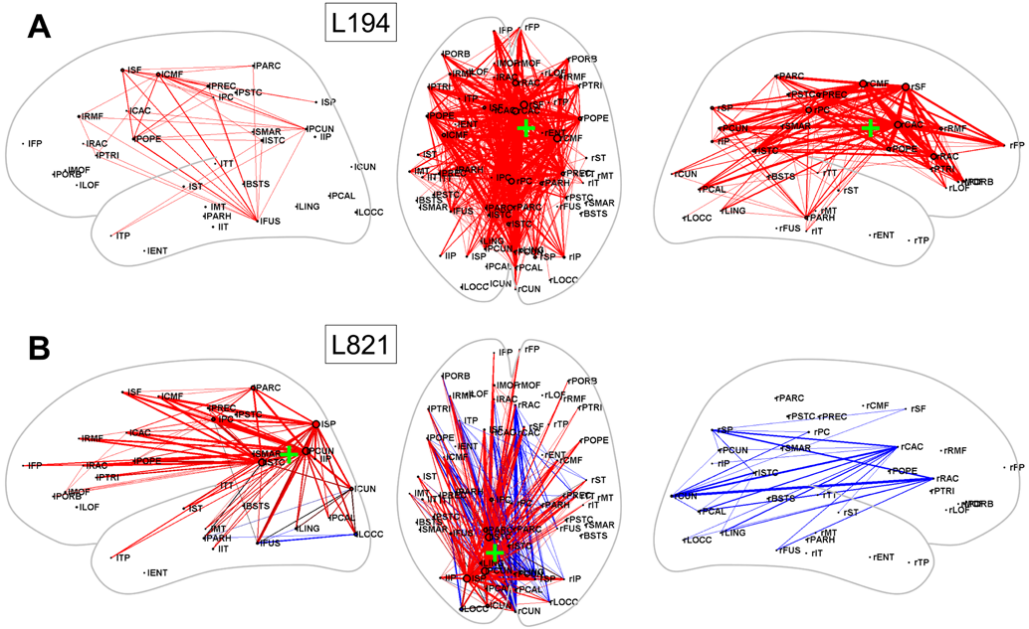
Dynamic effects of lesions along the brain's midline. (A) L194. (B) L821. In this plot, as well as in Figures 5, 6 and S1, a dorsal view of the brain (middle panel) and two lateral views of the left hemisphere (left panels) and the right hemisphere (right panels) are shown. The middle panel plots all significantly different functional connections, while the left and right panels only show significantly different functional connections within the left and right hemispheres, respectively. The 998 ROI z-score FC matrix was aggregated to 66 subregions, and pathways between these 66 subregions are plotted if at least 10% of their constituent connections linking ROI pairs are significantly changed (|z|>3.3) as a result of the lesion. Pathways are plotted in red or blue, if their coupling has been weakened or strengthened, respectively. The approximate lesion center is marked with a green “+”.

**Figure 5:**
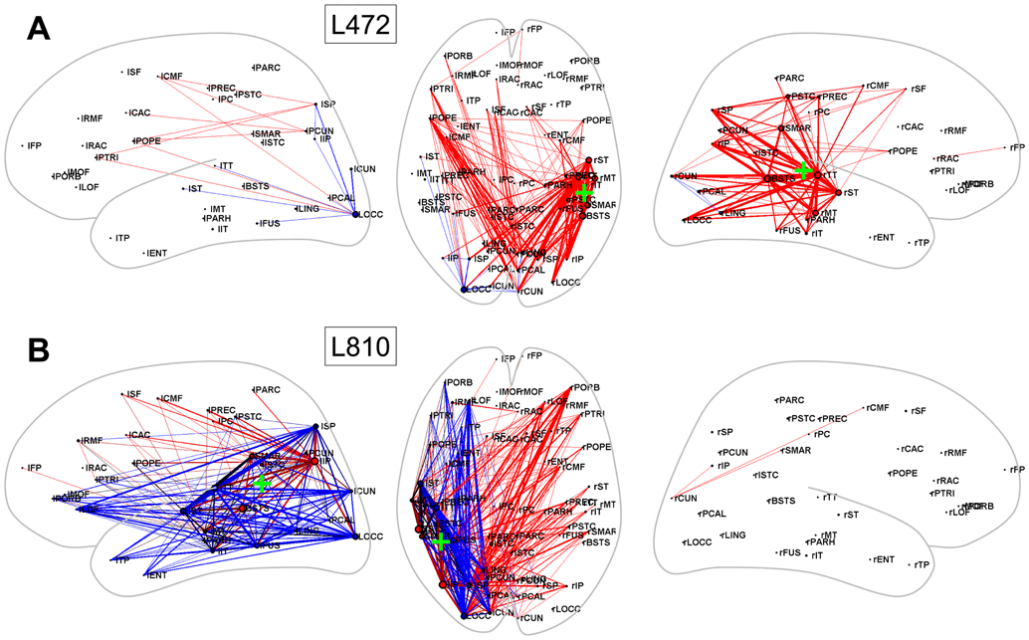
Dynamic effects of lesions near the temporo-parietal junction. (A) L472. (B) L810. For plotting conventions see legend to Figure 4.

**Figure 6:**
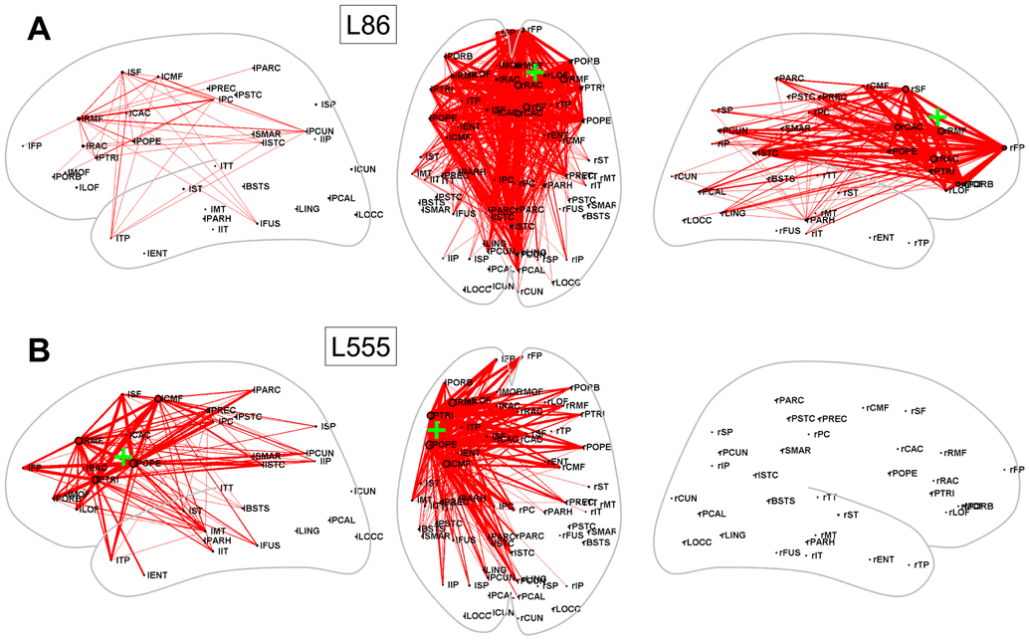
Dynamic effects of lesions in frontal cortex. (A) L86. (B) L555. For plotting conventions see legend to Figure 4.

Figures 4, 5 and 6 show the spatial distribution of functional connections that exhibited significant differences for a selection of lesion locations many of which were highly impactful overall, including lesions along the cortical midline (Figure 4), the temporo-parietal junction (Figure 5) and the frontal cortex (Figure 6). Other lesions altered functional connectivity less, for example lesions in primary sensory and motor regions (Figure S1).

Lesions along the cortical midline were characterized by widespread effects involving both cerebral hemispheres and all major cortical lobes. L194 (Figure 4A), centered in the right caudal anterior cingulate cortex resulted in lower functional connectivity between most ipsilateral subregions of right medial cortex, extending from orbitofrontal cortex to the cuneus. Some functional connections along the contralateral midline were also weakened, but to a lesser extent. Interhemispheric functional connections were profoundly suppressed. Lesions placed in the posterior medial cortex, e.g. L821 (Figure 4B) also affected functional connectivity in both hemispheres but had less widespread effects. Contralateral effects consisted of increasing coupling between several regions, including between superior parietal and anterior cingulate cortex.

Lesions near the temporo-parietal junction were highly disruptive of functional connectivity within their own cortical hemisphere as well as between hemispheres. L472 (Figure 5A) was centered in right superior temporal cortex and resulted in sharply lowered functional connectivity among all subdivisions of the ipsilateral parietal and posterior temporal cortex. In addition, coupling between regions in posterior medial cortex and frontal cortex were decreased in both hemispheres. A lesion in the left inferior parietal cortex in the vicinity of the left angular gyrus (L810, Figure 5B) significantly increased functional coupling within the left hemisphere, while suppressing cross-hemispheric functional connectivity.

Lesions involving parts of frontal cortex resulted in pronounced and widespread loss of functional coupling within the lesioned hemisphere as well as across hemispheres. A lesion of right superior frontal cortex (L86, Figure 6A) strongly reduced functional coupling of many right hemispheric brain regions, including interactions between frontal, temporal, and parietal cortex, extending over the entire length of the anterior-posterior axis. Weaker, but significant, suppression of functional connectivity is also seen in the contralateral hemisphere, including reduced coupling between the posterior cingulated/precuneus and the superior and middle frontal cortex. Lesioning left lateral frontal cortex centering on the pars opercularis (L555, Figure 6B) reduces functional coupling more locally.

Lesions of primary sensory and motor cortices (Figure S1) leave the functional connectivity of the remainder of the brain largely unchanged. Lesions centered in visual cortex (L360) or somatomotor cortex (L162) have little effect on functional connectivity outside of the immediate vicinity of the lesion itself.

In addition to node removal, lesions may be modeled as edge deletions, i.e. disruptions of white matter pathways. One of the most dramatic examples is the complete transection of the corpus callosum. We performed simulations after deleting all cross-hemispheric connections and compared the resulting functional connectivity patterns to those obtained from the intact brain (Figure S2). In the model, callosal transection resulted in the complete loss of all inter-hemispheric functional connectivity, as well as a more restricted pattern of significant changes in intra-hemispheric functional coupling.

**Figure 7:**
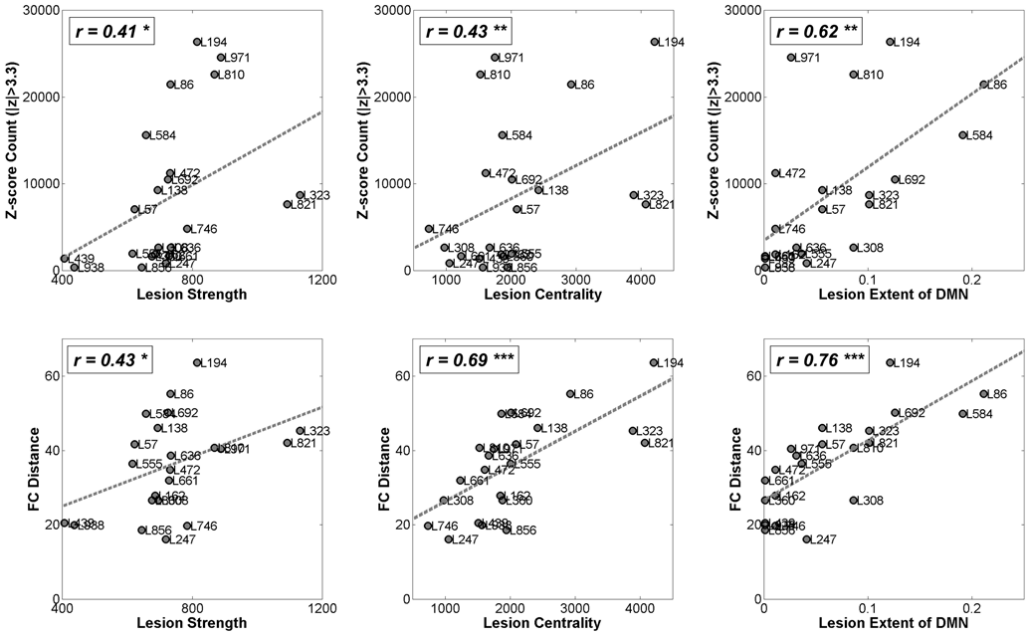
Summary diagram of relationships between structural lesion measures and dynamic lesion effects. Structural lesion measures are the sum of the node strengths of the lesion (“lesion strength”), the sum of the node centrality of the lesion (“lesion centrality”) and the extent to which the lesion included nodes within the DMN. Dynamic lesion effects are the number of significantly changed functional connections (outside of the lesioned nodes) and the distance between lesioned and unlesioned FC. Compare r-values to those in Table 3. * = p<0.05, ** = p<0.01, *** = p<0.001.

Finally, we examined whether the extent of dynamic lesion effects could be predicted on the basis of the impact of the lesion on structural network measures. Specifically, we asked if dynamic lesion effects were more pronounced if the lesion lengthened network paths, removed a larger number of long-range connections, or removed more highly connected or more highly central nodes. Table 3 and Figure 7 summarize the relationship between these structural measures and several measures of the dynamic impact of the lesion. The reported correlations are calculated for a subset of 22 lesion sites covering about 80 percent of the cortical surface, and for a single lesion size (50 nodes). The extent of dynamic lesion effects was only weakly predicted (r≈0.4–0.5) by the degree or strength of the nodes within each lesion. A better predictor was the number of connections between the lesion site and the rest of the brain (these connections are lost as a result of the lesion), and how much the lesion increased the path length of the remaining network (r≈0.45–0.7). Node and edge centrality of the lesioned nodes or edges predicted functional lesion impact about equally well (r≈0.45–0.7). The most robust prediction was made by the extent to which the lesion damaged the default mode network (r≈0.6–0.85).

**Table 3:** Magnitude of correlation between structural measures of the lesion and its dynamic effects.

		Structural Measure of Lesion						
		Degree	Strength	Fiber Count	Path Length	Node Centrality	Edge Centrality	DMN
Functional Measure of Lesion Effect	dFC(998)	0.4705 *	0.4253 *	0.6682 ***	0.6976 ***	0.6877 ***	0.6814 ***	0.7616 ***
	dFC′(998)	0.5364 *	0.5158 *	0.4872 *	0.4248 *	0.4237 *	0.4467 *	0.5979 **
	dFC (66)	0.2956 n.s.	0.2385 n.s.	0.5883 **	0.5759 **	0.5747 **	0.5956 **	0.8406 ***
	dFC′(66)	0.5168 *	0.4856 *	0.5461 **	0.4455 *	0.4484 *	0.4788 *	0.7095 ***
	z	0.5019 *	0.4562 *	0.6864 ***	0.7393 ***	0.7201 ***	0.7190 ***	0.7587 ***
	z′	0.4404 *	0.4136 *	0.4503 *	0.4503 *	0.4257 **	0.4519 *	0.6153 **

Structural measures are: Degree = sum of the degrees of the lesioned nodes; Strength = sum of the strengths of the lesioned nodes; Fiber Count = total number of all connections made between the lesioned nodes and the rest of the brain; Path Length = characteristic path length of the lesioned network; Node Centrality = sum of the centrality of all lesioned nodes; Edge Centrality = sum of the centrality of all lesioned edges; DMN = proportion of the DMN included in the lesion. Functional measures are: dFC(998) = distance between unlesioned and lesioned functional connectivity (998 nodes); dFC′(998) = same as dFC(998), but excluding all lesioned nodes; dFC(66), dFC′(66) = as before, for the low resolution (66 nodes); z = sum of all functional connections with |z|>3.3; z′ = same as z, but excluding functional connections of lesioned nodes. * = p<0.05, ** = p<0.01, *** = p<0.001, n.s. = non-significant.

## Discussion

The availability of whole-brain structural connectivity data sets [3]–[7], for the first time, allows for the computational study of the effects of localized structural lesions on neural dynamics. In this study, lesions are modeled as structural perturbations with specific dynamic effects. We find that lesions in different regions of the cerebral cortex have specific effects on the pattern of endogenous functional connectivity of the remaining brain that differ in both extent and spatial pattern. Generally, lesions along the cortical midline, the temporo-parietal junction and the frontal cortex result in the largest and most widespread effects on functional connectivity. Many lesions affect the functional coupling of brain regions outside of the lesion itself, including effects in the hemisphere contralateral to the lesion site.

The first part of our study involved random and targeted node deletions and their impact on the structural integrity of the network (Figure 3). Some of our results expanded upon observations made by other investigators who examined the vulnerability or robustness of brain networks [21]–[23]. Our structural network is relatively resilient against random node removal and against targeting of nodes on the basis of their high degree or high strength, a finding also reported for human functional networks [21]. However, the network is much less well protected against loss of nodes that are highly central, a finding that is consistent with the overall network architecture which consists of modules linked by hubs [4]. Targeted node removal by centrality may have a physiological basis. There is a potential link between node centrality and baseline metabolic activity [4] and it has been suggested that a high rate of metabolism may render neurons vulnerable to neurodegenerative processes [31],[32]. We hypothesize that at least some forms of degenerative brain disease may involve the “targeted” removal of network components.

Confirming earlier results obtained from a much smaller connection matrix of macaque cortex [24], modeling lesions in the human brain resulted in non-local dynamic effects. Several empirical studies have demonstrated such non-local effects, for example changes a distributed pattern of functional connectivity following in patients with focal brain lesions due to tumor or stroke [33]–[35]. Early theoretical accounts had predicted and attempted to explain such nonlocal effects, invoking concepts such as “diaschisis” [36] or “disconnection” [37]. The complex network approach adopted in this paper supports these concepts and provides a new opportunity to establish links between physical brain damage and functional disturbances. As suggested by studies of structural network measures [23],[38], including our own results regarding the effects of targeted node removal (Figure 3), we found that dynamic lesion effects were particularly large and widespread when lesions included nodes or edges of high centrality (Figure 7, Table 3). Another good predictor of functional change was the number of connections between the lesion site and the rest of the brain that were lost. This result underscores that “disconnection” may occur not only for areas that are directly anatomically linked but also account for changes in dynamic coupling among remote and structurally unconnected areas of cortex. Dynamic lesion effects were especially pronounced for several highly connected hub nodes within the brain's default mode network, for example in medial parietal and cingulate cortex. We believe that this result applies generally to the type of network and neural dynamics investigated here, and will hold even as the human connectome [39] continues to be refined.

The significant computational requirements involved in conducting large-scale simulations of endogenous brain activity necessitated we limit our analysis to a set of brain lesions selected for their neurological interest (Figure 1, Table 1). In the model, lesions of regions along the cortical midline were particularly disruptive. In patients, lesions of posterior medial cortex (in the vicinity of L323 and L821) are described as rare but resulting in profound disorders of consciousness [40], while lesions of the anterior cingulate cortex result in severe disruptions of personality and emotional processing, apathy and inattention [41]. In the model, lesions centered on the temporo-parietal junction also resulted in widespread changes in functional coupling. Empirically, the left angular gyrus (near L810) has been implicated in dyslexia [42], while lesions centered on the posterior portion of the right superior temporal cortex (near L472) often result in spatial hemineglect [43]. In contrast to these large effects of midline and temporo-parietal lesions, modeled lesions of primary visual and somatomotor cortex had little effect outside of their respective target regions. In patients, lesions of visual cortex or motor cortex result in deficits that are severe, but largely limited to loss of function within a specific modality. While our study does not provide complete coverage of all possible lesion sizes and locations in cortex we note that the magnitude and dispersion of the lesion's dynamic impact is correlated with the clinically observed severity and range of cognitive deficits.

In the current model we did not attempt to include the effects of lesions of brain nodes on white matter “fibers of passage”, and neither did we attempt to systematically explore the functional impact of disruptions of specific white matter pathways. We provided a single example of fiber damage by modeling the effects of cutting all inter-hemispheric connections (Figure S2). The observed pattern matches empirical observations of a striking loss of inter-hemispheric functional connectivity immediately following callosotomy in a human patient [29]. Contrasting this observation, residual functional connectivity between the two cerebral hemispheres observed in a patient several decades after a complete commissurotomy [44] may be due to inter-hemispheric coupling via subcortical pathways. An extension of the current model to include subcortical nodes and connections may provide a structural basis for the long-term restoration of inter-hemispheric functional connectivity following callosal transection.

At the present stage, the model cannot be tested for behavioral or cognitive deficits. While future studies may include a quantitative evaluation of the structure of pre-/post-lesion effective brain networks resulting from specific task-related perturbations, here we relied exclusively on the pattern of endogenous neural dynamics to measure dynamic lesion impact. These endogenous dynamics may be viewed as a proxy for the cortical “resting state” in the human brain [45],[46], which has been shown to be disrupted or altered in the course of disease states [31]. For example, changes in resting-state activation and functional connectivity may serve as diagnostic markers for the onset, progression or severity of Alzheimer's disease [32] and schizophrenia [47],[48]. Both conditions are known to be associated with disturbances of structural brain connectivity, including portions of the default mode network. Here, we observed that lesions that included portions of the default mode network had particularly large and widespread effects on functional connectivity throughout the brain. This is consistent with previously observed strong structural and functional coupling among ROIs in the DMN [8] and its association with major hubs in the cortex [4],[32]. Our model suggests that the pattern of endogenous neural activity, in particular within the default mode network, may serve as a marker of the degree of functional disturbance. A further implication is that the restoration of the topology of resting-state functional connectivity may aid in cognitive repair and recovery [49].

The structural connectivity pattern used in the present model was obtained by noninvasive diffusion imaging [4]. Future mapping studies of the human connectome will likely provide improved imaging and reconstruction of crossing, highly curved, or long-distance fiber pathways, thus providing a more accurate structural model. In addition, several current limitations of the model should be addressed: a) The model contains only cerebral cortical regions and pathways, and does not account for axonal conduction delays; b) The model does not take into account white matter damage of “fibers of passage” in addition to node deletion; c) The model captures only immediate lesion effects without including mechanisms of neural plasticity which may support reorganization and functional recovery. We believe these limitations can be overcome as available data sets and computational modeling tools improve. A particularly fruitful avenue for future work is the incorporation of longitudinal data on structural and functional processes following acute brain injury. The further development of noninvasive imaging technology in combination with sophisticated computational modeling may eventually allow the design of individualized treatment and recovery protocols that help improve behavioral outcomes following acute cortical lesions.

## Supporting Information

Figure S1 Dynamic effects of lesions in primary sensory and motor regions. For plotting conventions see legend to Figure 4 (main text).
(1.55 MB TIF)

Figure S2 Dynamic effects of the complete transection of all interhemispheric connections (corpus callosum). The panel on the left shows the intact pattern of functional connectivity, estimated from a seed region located near the right hemispheric frontal eye fields at [28, -7, 54], matching the seed location in Figure 2 of ref. [29]. The intact pattern shows positive coupling between frontal and parietal cortex, as well as between homologous structures in the two hemispheres. The panel on the right shows the pattern of functional connectivity, again seeded at [28, -7, 54], after complete transection of all callosal connections. Interhemispheric functional connections are abolished, while intrahemipsperic functional connections are largely preserved.
(5.05 MB TIF)
