# Supplementary figures and images for: Modeling the Impact of Lesions in the Human Brain
Source: PLoS Comput Biol. 2009 Jun 12;5(6):e1000408. doi: 10.1371/journal.pcbi.1000408 (PMC2688028; doi:10.1371/journal.pcbi.1000408)

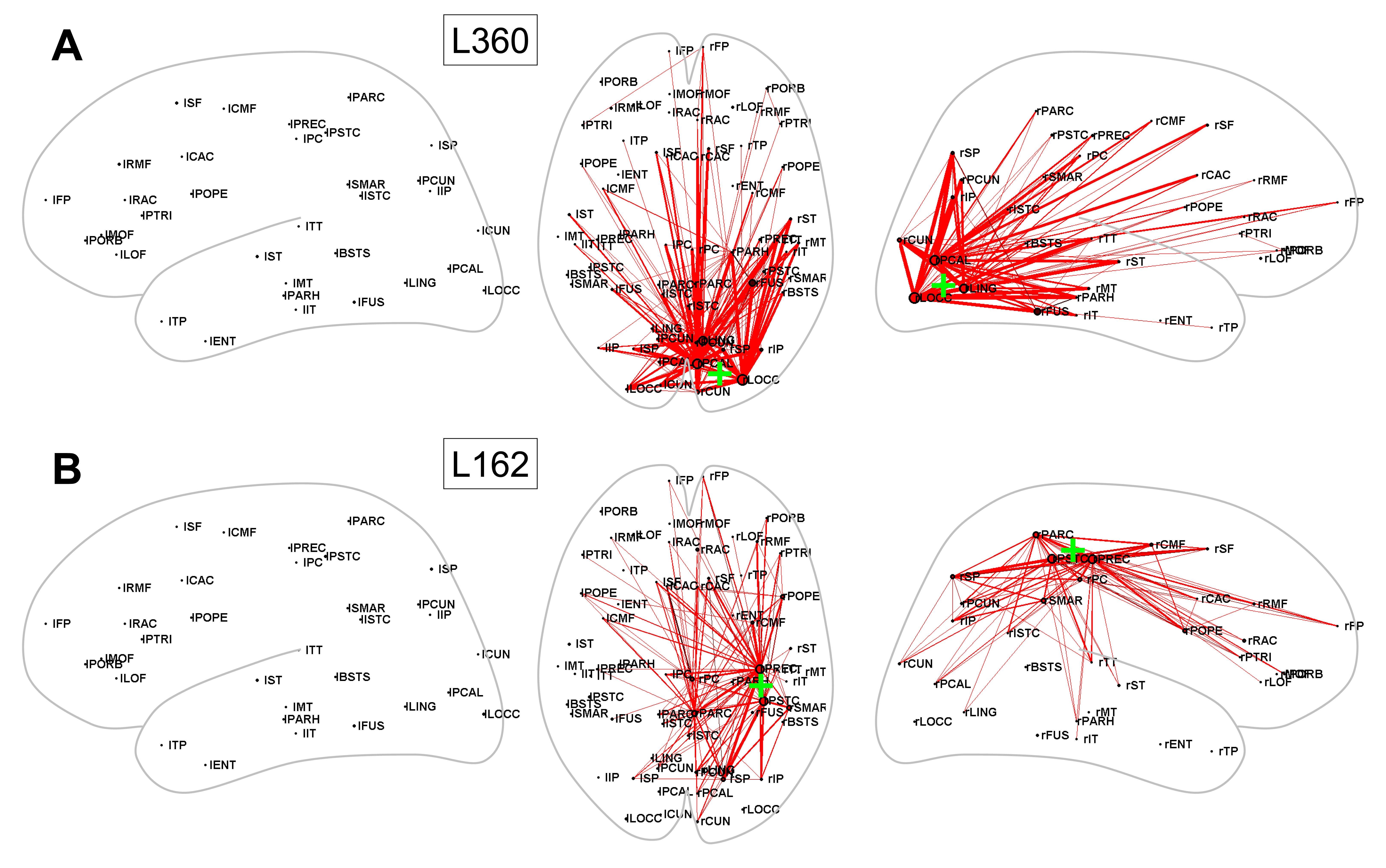

Supplement: Figure S1 — Dynamic effects of lesions in primary sensory and motor regions. For plotting conventions see legend to Figure 4 (main text). (1.55 MB TIF) [file pcbi.1000408.s001.tif]

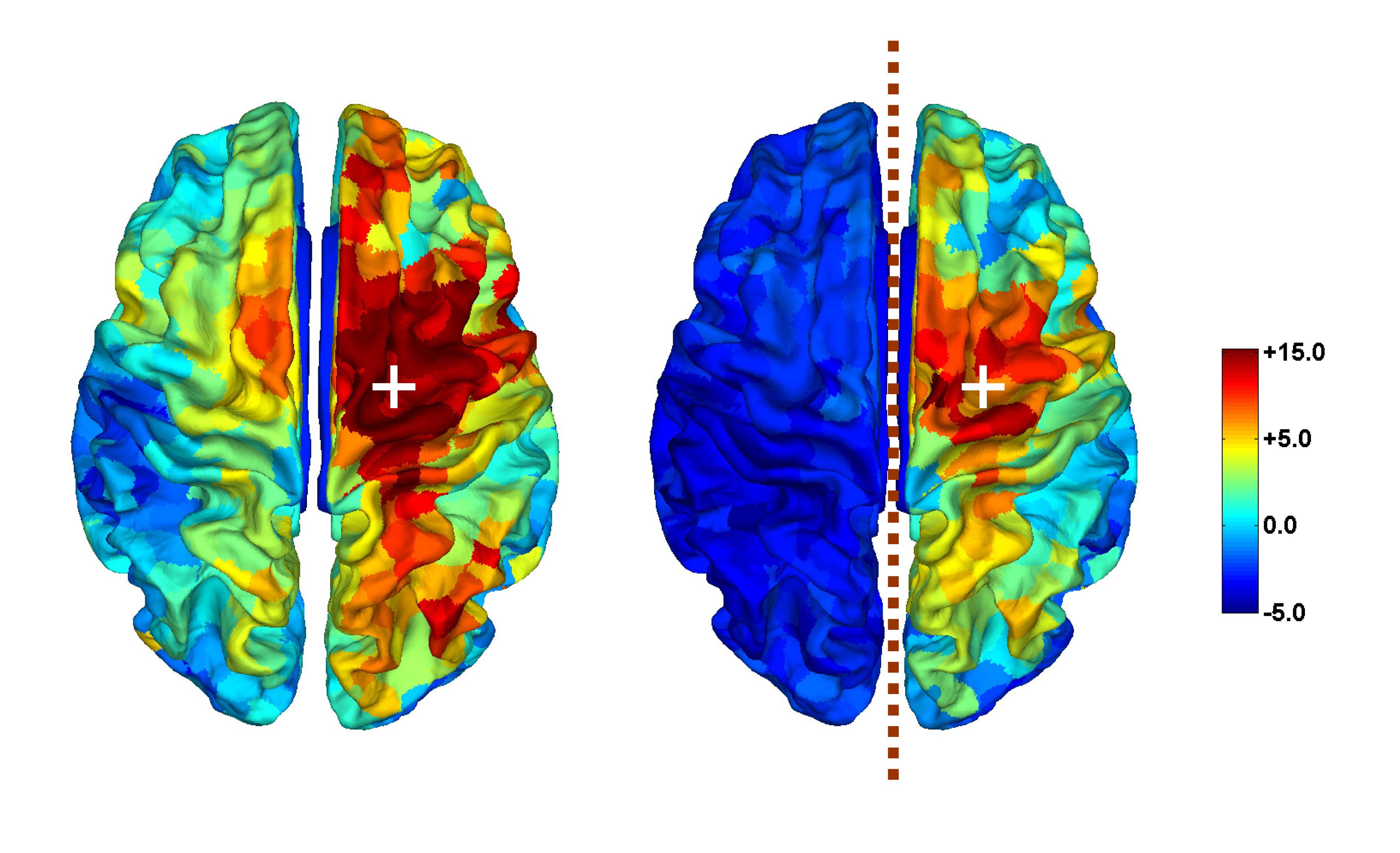

Supplement: Figure S2 — Dynamic effects of the complete transection of all interhemispheric connections (corpus callosum). The panel on the left shows the intact pattern of functional connectivity, estimated from a seed region located near the right hemispheric frontal eye fields at [28, -7, 54], matching the seed location in Figure 2 of ref. [29]. The intact pattern shows positive coupling between frontal and parietal cortex, as well as between homologous structures in the two hemispheres. The panel on the right shows the pattern of functional connectivity, again seeded at [28, -7, 54], after complete transection of all callosal connections. Interhemispheric functional connections are abolished, while intrahemipsperic functional connections are largely preserved. (5.05 MB TIF) [file pcbi.1000408.s002.tif]
